# Safety, tolerability of ES16001, a novel varicella zoster virus reactivation inhibitor, in healthy adults

**DOI:** 10.1186/s40001-021-00565-z

**Published:** 2021-08-12

**Authors:** Jeon Hwang-Bo, Byungwook Kim, Dae Won Park, Yeong-Geun Lee, Jeong Eun Kwon, Jae-Yong Chung, Se Chan Kang

**Affiliations:** 1 Graduate School of Biotechnology and Department of Oriental Medicine Biotechnology, Kyung Hee University, Yongin, 17104 Republic of Korea; 2 Department of Clinical Pharmacology and Therapeutics, Seoul National University College of Medicine and Hospital, Seoul, Republic of Korea; 3 Department of Clinical Pharmacology and Therapeutics, Seoul National University College of Medicine and Bundang Hospital, Seongnam, Republic of Korea

**Keywords:** *Elaeocarpus sylvestris*, ES16001, Safety and tolerability, Varicella zoster virus

## Abstract

**Purpose:**

Herpes zoster (HZ), or shingles, is a clinical syndrome resulting from the reactivation of latent varicella zoster virus (VZV) within the sensory ganglia. We evaluated the safety and tolerability of ES16001 (ethanol extract of *Elaeocarpus sylvestris *var.* ellipticus*), a novel inhibitor of varicella zoster virus reactivation in healthy adults.

**Method:**

Single-center, randomized, double-blind, placebo-controlled, single and multiple ascending dose (SAD and MAD, respectively) studies were conducted in 20- to 45-year-old healthy adults without chronic disease. In the SAD study (*n* = 32), subjects randomly received a single oral dose of 240, 480, 960, or 1440 mg ES16001 or a placebo. In the MAD study (*n* = 16), subjects randomly received once daily doses of 480 or 960 mg ES16001 or a placebo for 5 days. The safety and tolerability of the drug were evaluated by monitoring participants’ treatment emergent adverse events (TEAEs) and vital signs, electrocardiograms (ECGs), physical examinations, and clinical laboratory tests.

**Results:**

In the SAD study, 11 adverse reactions were seen in 5 subjects, and in the MAD study, 8 adverse reactions were seen in 6 subjects. All adverse reactions were mild, and no serious adverse reactions occurred. The most common adverse reaction was an increase in alanine aminotransferase (ALT), but all test values were in the clinically non-significant range, and their clinical significance was judged to be small considering the fact that most of the test values returned to normal immediately after the end of drug administration.

**Conclusion:**

ES16001 has good safety and tolerability when administered both once and repeatedly to healthy subjects. Further research is needed to identify any possible drug-induced hepatotoxicity, which appears infrequently. Our findings provide a rationale for further clinical investigations of ES16001 for the prevention of HZ.

*Trial registration*: CRIS, KCT0006066. Registered 7 April 2021—Retrospectively registered, https://cris.nih.go.kr/cris/search/detailSearch.do/19071).

**Supplementary Information:**

The online version contains supplementary material available at 10.1186/s40001-021-00565-z.

## Introduction

Varicella zoster virus (VZV) is generally a childhood infection and causes chickenpox. It moves along the entire nerve axis in the body and hides in the dorsal root ganglion, cranial ganglion, and autonomic ganglion, and can reactivate when the host's immunity decreases, causing shingles [1]. This clinical form of shingles is characterized by a painful, unilateral vesicular eruption, which usually occurs in a restricted dermatomal distribution [2]. Although herpes zoster can occur at any age, it is mainly a disease of adults > 60 years of age. The recurrence rate of shingles is reported to be about 5% or more; it is a difficult disease to treat because existing shingles treatments cannot block the reactivation of the virus latent in the ganglion [3]. Therefore, inhibition of VZV reactivation is known to be an important factor in the treatment of shingles.

Currently, antiviral drugs for the treatment of VZV-associated diseases include acyclovir (ACV), valacyclovir, famciclovir/penciclovir, and foscarnet that aim to inhibit viral DNA polymerase [4, 5]. While these antiviral drugs are effective and relatively safe, a number of adverse reactions are associated with their use such as headache, vomiting, diarrhea, nausea, phlebitis, and abdominal pain. Furthermore, VZV strains resistant to these antiviral drugs have been reported, emphasizing the importance of developing novel therapeutic agents for VZV-associated diseases [6, 7]. However, no other agents have been developed so far, and current treatments cannot block the reactivation of VZV, which is latent in the ganglion.

*Elaeocarpus sylvestris var. ellipticus* (ES) is a genus of tropical and subtropical evergreen trees and shrubs. Its region of distribution includes the subtropical zone from Jeju in Korea to southern China, Okinawa, Kyushu (Japan), and Taiwan [8]. ES is used as a landscape tree and is fast-growing and easy to breed with strong adaptability [9]. A recent study reported that a 70% ethanol extract of ES inhibits the replication of human cytomegalovirus (HCMV), which causes megacytosis, in vitro [10]. In addition to its anti-HCMV effect, the ethanol extract of ES has been reported to possess antioxidant activity and protect mice from gamma-ray-induced immunosuppression [11, 12]. In our previous study, a 50% ethanol extract (ESE) of ES inhibited the expression of VZV replication-related genes and cell death due to viral infection. It also has an inhibitory effect on peripheral and central inflammatory pain that can be caused by herpes [13]. In addition, we confirmed the effect of ESE on the activity of VZV through hollow fiber assay using VZV-infected Vero cells. Further, cells and viruses obtained in the hollow fiber assay were treated in cultured Vero cells to confirm the effect of ESE on VZV reactivation. For the quantification of VZV, viral DNA was extracted and qRT-PCR was used to measure the expression level of the ORF38 gene, which is involved in the viral replication process. We determined that the activity of VZV was greatly suppressed in the ESE-treated group. Particularly, in the group administered 50 mg/kg and 100 mg/kg ESE, the reactivation rates were − 4.2% and − 60.4%, respectively, indicating that ESE inhibited the reactivation of VZV (Additional file 1: Table S1). These data suggest that ES16001 might be clinically more effective than existing treatments, providing a rationale for the clinical development of this drug. Therefore, in this clinical trial, we evaluated the safety and tolerability of ES16001 administered by single or repeated oral administration to healthy adult subjects.

## Materials and methods

This clinical trial (GNC_PT1_2018001) was reviewed and approved by the Institutional Review Board of Seoul National University Bundang Hospital (B-1908/559-002) and was performed in accordance with Korean Good Clinical Practice (KGCP). This clinical study is registered in the Clinical Research Information Service of the Republic of Korea (CRIS No. KCT0006066). This study is based on the Fortaleza’s Declaration of Helsinki in 2013 and was conducted with the rights and welfare of the subjects in mind. Prior to screening, all subjects provided written informed consent to participate in the study after having been informed about the nature and purpose of the study and participation/withdrawal conditions.

### Study drug

ES16001 is a tablet consisting of 50% ethanol extract of ES, one tablet of ES16001 40 mg contains 40 mg of 50% ethanol extract of ES out of a total of 429 mg. One tablet of ES16001 80 mg and 120 mg contains 80 mg and 120 mg of ES 50% ethanol extract, respectively, out of a total of 429 mg. The ES16001 for oral administration and placebo tablets was supplied by Genencell Co., Ltd. (Yongin, Gyeonggi-do, Korea). Placebo tablets were identical to the ES16001 tablets (a green, rectangular, film-coated tablet) in size, texture, and color. The manufacturing process was in accordance with the good manufacturing process (GMP) standards, and the drugs were delivered and stored according to the manufacturer’s instructions.

### Study population

This study included healthy adults 20 to 45 years old without chronic disease, weighing 55 kg or more, and weighing within ± 20% of ideal body weight (IBW):

$${\text{Ideal body weight }}\left( {{\text{IBW}}} \right) \, = \, \left( {{\text{height }}\left( {{\text{cm}}} \right) \, {-}{ 1}00} \right) \, \times \, 0.{9}{\text{.}}$$

After a total of 87 subjects agreed in writing, screening tests including demographic information verification, vital signs and physical examination, clinical laboratory tests, serum tests, urine tests, ECG, pregnancy tests, and concomitant drug identification were conducted on subjects who agreed.

**Fig. 1:**
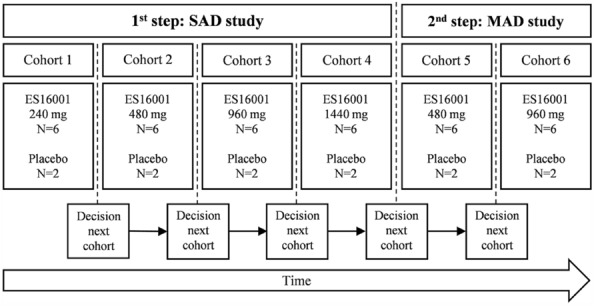
Trial flowchart

In the SAD study, 33 subjects passed the inclusion and exclusion criteria, and 8 subjects (ES16001—6 subjects, placebo—2 subjects) were randomly assigned to each dose group. One subject withdrew consent before drug administration due to personal reasons. Finally, 32 subjects (24 subjects in the ES16001 group, 8 subjects in the placebo group) completed the clinical trial and were included in the safety/tolerability evaluation. In the MAD study, 18 subjects passed the inclusion/exclusion criteria, and 8 subjects (ES16001 6 subjects, placebo 2 subjects) were randomly assigned to each dose group. Two subjects withdrew consent before drug administration due to personal reasons. Finally, 16 subjects (12 subjects in the study group, 4 subjects in the placebo group) completed all clinical trials (Fig. 1) and were included in the safety/tolerability evaluation. All subjects ended the study according to the clinical trial protocol, and none of the subjects had any significant protocol violations.

### Study design

**Fig. 2:**
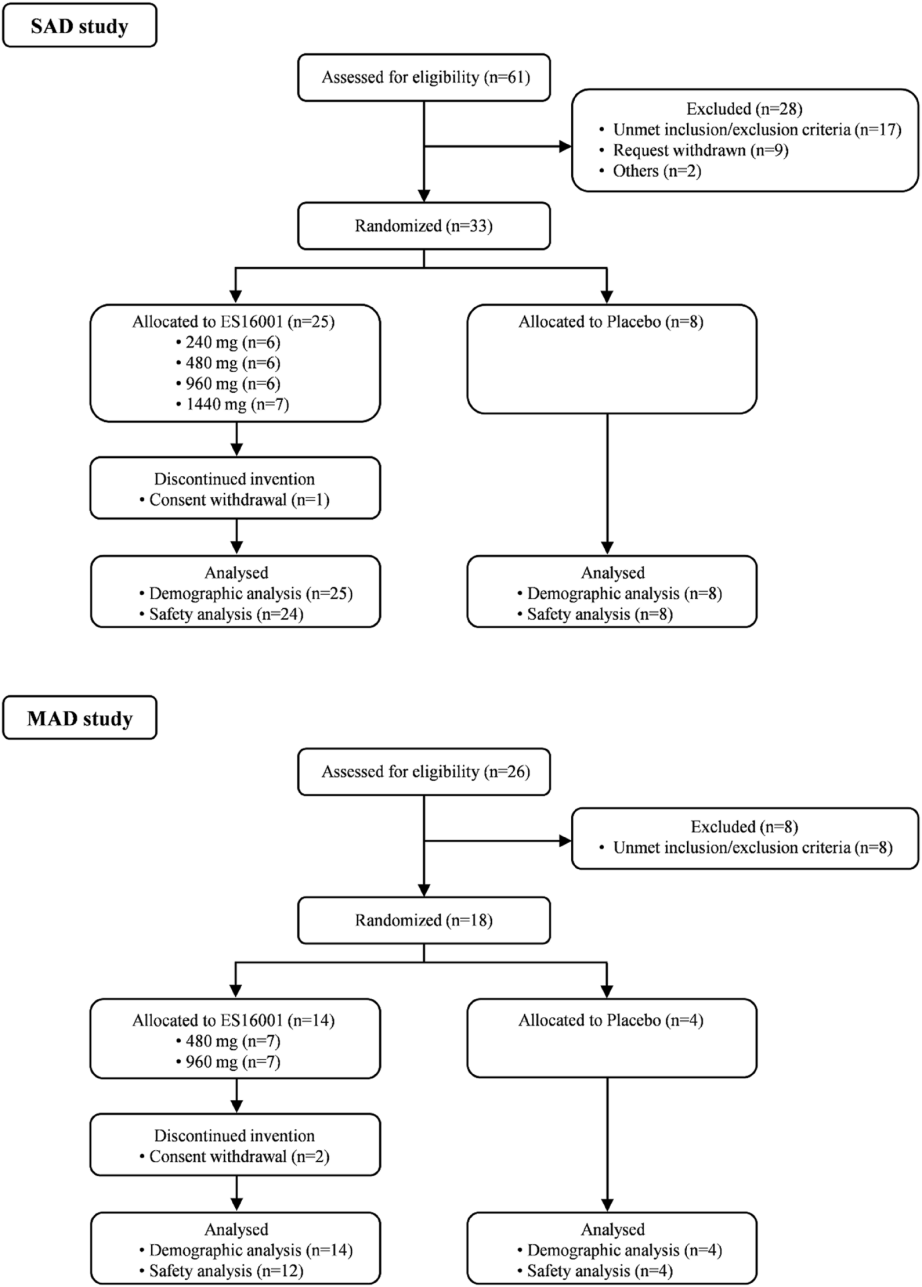
Study outline

This clinical trial consisted of a single-center, randomized, double-blind, placebo-controlled, single and multiple ascending dose trial. In the SAD study, four dose groups (240, 480, 960, and 1440 mg/day) were administered once to confirm safety, and in the MAD study, two dose groups (480 and 960 mg/day) were repeatedly administered for 5 days to confirm safety. The dose was increased sequentially from the low-dose group, and after analyzing the safety and tolerability results in the first-stage SAD study, the second-stage MAD study was begun. In the case of the SAD study, safety data (clinical laboratory tests, vital signs, ECG tests, adverse reactions, etc.) were reviewed for all subjects in each dose group from immediately after administration to 8 days after administration. In the case of the MAD study, safety data from immediately after dosing to 12 days after initial administration for all subjects in each dose group were reviewed. If the data indicated that a dose was safe, the next higher incremental dose was studied in the subsequent cohort. In the SAD study, cohort 1 participants received ES16001 240 mg or placebo, cohort 2 participants received ES16001 480 mg or placebo, cohort 3 participants received ES16001 960 mg or placebo, and cohort 4 participants received ES16001 1,440 mg or placebo; the ES16001 or placebo was administered orally once. In the MAD study, cohort 5 participants received ES16001 480 mg/day or placebo and cohort 6 participants received ES16001 960 mg/day or placebo daily for 5 days (Fig. 2).

### Safety evaluation

The safety assessment included all subjects who received one or more doses of the clinical trial drug. Analysis was performed according to the administration group and treatment group of the clinical trial drug, and all adverse reactions occurred during the clinical trial were recorded. All adverse reactions were standardized in System Organ Class (SOC) and Preferred Term (PT) based on the Medical Dictionary for Regulatory Activities (MedDRA, version 23.0). The frequency (N), percentage (%) and number of adverse reactions (F) and adverse drug reactions that occurred in this study are presented based on SOC and PT according to the dose group, the treatment group, and severity of the reaction. If there were significant changes in the clinical laboratory test (hematology test, blood chemistry test, blood coagulation test, urine test) values, vital signs test values (systolic blood pressure, diastolic blood pressure, heart rate, body temperature), and 12-lead ECG test values (ventricular rate, PR interval, QRS duration, QT interval, QTc interval) of any subject, they were described by the dose group. If each outcome was a clinically significant change, it was determined whether it was related to the clinical trial drug.

### Statistical analysis

Statistical analyses were performed on all subjects who received one or more administration of the clinical trial drug. Continuous results are presented for each randomized dose group using descriptive statistics (number of subjects, average, standard deviation, coefficient of variation, minimum, median, and maximum) for analysis, and categorical results are presented by frequency and percentage. The values measured at the closest time prior to the administration time were used as baseline data. Outliers of the data were excluded and further analyzed at the decision of the examiner. Statistical analyses were performed using verified software (SAS Ver 9.4, Cary, NC, USA).

## Results

### Demographic characteristics of subjects

In the SAD study, a total of 33 subjects of 61 volunteers who received the screening test were enrolled in the clinical trial. In the MAD study, a total of 18 subjects of 26 volunteers who received the screening test were enrolled in the clinical trial. Subsequently, 6 subjects were randomly assigned to the treatment group of each administration group, and 2 subjects were randomly assigned to the placebo group. All subjects were male. In the SAD study, the demographic characteristics of the subjects were age 26.42 ± 3.92 years old, height 174.13 ± 5.08 cm, weight 71.28 ± 8.04 kg, body mass index (BMI) 23.45 ± 1.89 kg/m^2^, and IBW 66.72 ± 4.57 kg (Table 1). In the MAD study, demographic characteristics of the subjects were age 26.89 ± 6.70 years old, height 175.01 ± 4.99 cm, weight 71.78 ± 7.76 kg, BMI 23.41 ± 2.05 kg/m^2^, and IBW 67.52 ± 4.48 kg (Table 2). There were no statistically significant differences in the demographic characteristics of subjects assigned to each dose group and placebo group, excluding smoking history (*p*-value: 0.0234, Fisher's exact test) (Table 2).

**Table 1:** Demographic characteristics of intention-to-treat population: SAD study

Parameters	Dose group					Total (*N* = 33)	*p*-value
	240 mg (*N* = 6)	480 mg (*N* = 6)	960 mg (*N* = 6)	1440 mg (*N* = 7)	Placebo (*N* = 8)		
Age (years)	26.50 ± 5.75	27.67 ± 3.98	26.83 ± 1.83	26.14 ± 3.02	25.38 ± 4.72	26.42 ± 3.92	0.7707
Height (cm)	173.40 ± 7.34	173.75 ± 4.38	172.03 ± 3.86	176.37 ± 3.90	174.56 ± 5.70	174.13 ± 5.08	0.6516
Weight (kg)	74.50 ± 10.44	72.75 ± 6.99	65.63 ± 9.08	74.56 ± 6.43	69.11 ± 5.84	71.28 ± 8.04	0.1871
BMI (kg/m^2^)	24.67 ± 1.87	24.05 ± 1.15	22.10 ± 2.15	23.94 ± 1.46	22.70 ± 1.95	23.46 ± 1.90	0.0777
IBW (kg)	66.05 ± 6.60	66.40 ± 3.93	64.85 ± 3.47	68.73 ± 3.51	67.10 ± 5.13	66.72 ± 4.57	0.6516
Alcohol (n, %)	3 (50.0)	4 (66.7)	1 (16.7)	3 (42.9)	6 (75.0)	17 (51.5)	0.2748
Smoking (n, %)	4 (66.7)	3 (50.0)	3 (50.0)	3 (42.9)	2 (25.0)	15 (45.5)	0.6525

*N* number of subject

*p*-values by Kruskal–Wallis test (age, height, weight, BMI, and IBW)

*p*-values by Fisher’s exact test (alcohol, smoking)

BMI (kg/m^2^) = weight (kg)/{height (m)}^2^

Data presented as *N* (%) (alcohol, smoking)

**Table 2:** Demographic characteristics of intention-to-treat population: MAD study

Parameters	Dose group			Total (*N* = 18)	*p*-value
	480 mg (*N* = 7)	960 mg (*N* = 7)	Placebo (*N* = 4)		
Age (years)	28.43 ± 6.24	23.00 ± 1.29	31.00 ± 10.49	26.89 ± 6.70	0.0968
Height (cm)	176.80 ± 5.15	175.01 ± 3.46	171.88 ± 6.62	175.01 ± 4.99	0.2509
Weight (kg)	72.96 ± 9.36	70.99 ± 5.42	71.10 ± 10.09	71.78 ± 7.76	0.6339
BMI (kg/m^2^)	23.30 ± 2.54	23.19 ± 1.83	23.98 ± 1.93	23.41 ± 2.05	0.8855
IBW (kg)	69.13 ± 4.63	67.53 ± 3.10	64.70 ± 5.94	67.52 ± 4.48	0.2509
Alcohol (*n*, %)	5 (71.4)	2 (28.6)	4 (100)	11 (61.1)	0.0608
Smoking (*n*, %)	6 (85.7)	1 (14.3)	1 (25.0)	8 (44.4)	0.0235

*N* number of subject

*p*-values by Kruskal–Wallis test (age, height, weight, BMI, and IBW)

*p*-values by Fisher’s exact test (alcohol, smoking)

BMI (kg/m^2^) = weight (kg)/{height (m)}^2^

Data presented as *N* (%) (alcohol, smoking)

### Safety and tolerability

In the SAD study, a total of 11 adverse reactions (240 mg: 4 cases/1 subject, 480 mg: 1 case/1 subject, 960 mg: 2 cases/1 subject, 1440 mg: 2 cases/1 subject, placebo: 1 cases/1 subject) in 5 of 32 subjects received at least one clinical trial drug or placebo. All adverse reactions were mild, and ADRs from the SAD study are summarized in Table 3. The ADR for which the causality with the clinical trial drug was evaluated as ‘probably related’ was 1 case/1 subject (alanine aminotransferase increased) in the 240 mg dose group and 2 cases/1 subject (aspartate aminotransferase increased, alanine aminotransferase increased) in the 1440 mg dose group. The ADR evaluated as ‘possibly related’ was 1 case/1 subject (abdominal discomfort) in the 480 mg dose group. Finally, the number of ADRs evaluated as ‘probably not related’ was 7 cases/4 subjects in all dose groups. There was no significant difference (*p*-value = 1.0000, Fisher’s exact test) between the dose groups in the proportion of subjects with more than one adverse reaction judged to have a causality with the clinical trial drug. The most common ADR was an increase in alanine aminotransferase, which occurred in 2 cases/2 subjects. Increases in AST and ALT (AST range: 36–108, ALT range: 58–210) were observed in 1 subject in the 1440 mg dose group; symptoms occurred on Day 14 and were followed up to Day 35. Because symptoms persisted, on Day 42, a gastroenterology examination was conducted. Ursodeoxycholic acid and silymarin were prescribed, and the AST/ALT levels recovered to the normal range after six follow-up visits by the gastroenterologist over 144 days. This case was suspected drug-induced liver injury (DILI) because other abnormal causes cannot be found in the medical examination findings. There were no abnormal findings or changes with clinical significance as a result of clinical laboratory examination, vital signs, physical examination, or ECG.

**Table 3:** Summary of ADRs by treatment, SOC, and PT: SAD study

SOC and PT	Dose group					Total (*N* = 32) *n* (%) [F]
	240 mg (*N* = 6) *n* (%) [F]	480 mg (*N* = 6) *n* (%) [F]	960 mg (*N* = 6) *n* (%) [F]	1440 mg (*N* = 6) *n* (%)[F]	Placebo (*N* = 8) *n* (%)[F]	
Subjects with AEs	1 (16.7) [4]	1 (16.7) [1]	1 (16.7) [2]	1 (16.7) [3]	1 (12.5) [1]	5 (15.6) [11]
Fever			1 (16.7) [1]			1 (3.1) [1]
Fever			1 (16.7) [1]			1 (3.1) [1]
Nervous system disorders					1 (12.5) [1]	1 (3.1) [1]
Headache					1 (12.5) [1]	1 (3.1) [1]
General disorders and administration site conditions	1 (16.7) [2]					1 (3.1) [2]
Pyrexia	1 (16.7) [1]					1 (3.1) [1]
Chills	1 (16.7) [1]					1 (3.1) [1]
Investigations						2 (6.3) [4]
c-Reactive protein increased	1 (16.7) [2]			1 (16.7) [2]		1 (3.1) [1]
Aspartate aminotransferase increased	1 (16.7) [1]			1 (16.7) [1]		1 (3.1) [1]
Alanine aminotransferase increased	1 (16.7) [1]			1 (16.7) [1]		2 (6.3) [2]
Gastrointestinal disorders				1 (16.7) [1]		2 (6.3) [2]
Diarrhea		1 (16.7) [1]		1 (16.7) [1]		1 (3.1) [1]
Abdominal discomfort		1 (16.7) [1]				1 (3.1) [1]
Upper respiratory infection			1 (16.7) [1]			1 (3.1) [1]
Upper respiratory infection			1 (16.7) [1]			1 (3.1) [1]

Dictionary: MedDRA 23.0

SOC: System Organ Class and PT: Preferred Term

Subject is counted once at the maximum intensity if the subject reported one or more events

Percentages are based on the subjects within each treatment

In the MAD study, a total of 8 adverse reactions (480 mg: 6 cases/5 subject, 960 mg: 2 cases/1 subject) occurred in 6 of 16 subjects who received more than one dose of the clinical trial drug. All adverse reactions were TEAEs that occurred after administration of the clinical trial drug, and all adverse reactions were classified as ADR because the causality with the clinical trial drug could not be excluded. All adverse reactions were mild, and ADRs from the MAD study are summarized in Table 4.

**Table 4:** Summary of ADRs by treatment, SOC, and PT: MAD study

SOC and PT	Dose group			Total (*N* = 32) *n* (%) [F]
	480 mg (*N* = 6) *n* (%) [F]	960 mg (*N* = 6) *n* (%) [F]	Placebo (*N* = 8) *n* (%) [F]	
Subjects with AEs	5 (83.3) [6]	1 (16.7) [2]		6 (37.5) [8]
Investigations	4 (66.7) [4]			4 (25.0) [4]
Blood triglycerides increased	1 (16.7) [1]			1 (6.3) [1]
Blood creatine phosphokinase increased	3 (50.0) [3]			3 (18.8) [3]
Gastrointestinal disorders		1 (16.7) [2]		1 (6.3) [2]
Diarrhea		1 (16.7) [1]		1 (6.3) [1]
Abdominal pain		1 (16.7) [1]		1 (6.3) [1]
Skin and subcutaneous tissue disorder	1 (16.7) [2]			1 (6.3) [2]
Hyperhidrosis	1 (16.7) [1]			1 (6.3) [1]
Pruritus	1 (16.7) [1]			1 (6.3) [1]

Dictionary: MedDRA 23.0

SOC: System Organ Class and PT: Preferred Term

Subject is counted once at the maximum intensity if the subject reported one or more events

Percentages are based on the subjects within each treatment

There were 2 cases/1 subject for which the causality of the ADRs with (diarrhea, abdominal pain) the clinical trial drug was evaluated as ‘probably related’ in the 960 mg dose group. In 6 cases/5 subjects, the ADRs were evaluated as ‘probably not related’ (blood triglycerides increased 1 case, blood creatinine phosphokinase increased 3 cases, hyperhidrosis 1 case, pruritus 1 case) in all dose groups. No ADR occurred in the MAD placebo group. There was a significant difference (*p*-value = 0.0235, Fisher’s exact test) between dose groups in the proportion of subjects who had one or more adverse reactions (ADR evaluated as ‘probably not related’ or higher) judged to have a causality with the clinical trial drug. The most common ADR was an increase in blood creatinine phosphokinase, for which 3 cases occurred in 3 subjects. One of the subjects with increased blood creatinine phosphokinase also had an increase in their baseline CPK level to 360 IU/L, higher than the normal level, and all other subjects with increased blood creatinine phosphokinase had their CPK levels reached their maximum on Day 7. But there was no statistically significant difference compared to pre-dose CPK levels.

Descriptive statistical analyses of AST and ALT levels determined that these compounds had a tendency to decrease in most of the administration groups after reaching the maximum on Day 7. Statistical analysis of liver function tests showed a statistically significant increase in ALT in the 480 mg administration group. However, all test values were in the clinically insignificant range, and clinical significance was judged to be small based on the fact that most of them returned to normal values immediately after the end of the administration. There were no abnormal findings or changes with clinical significance as a result of clinical laboratory examination, vital signs, physical examination, or ECG.

Taken together, these results demonstrate that in healthy volunteers ES16001 has good safety and tolerability in the range of doses from 240 mg to 1440 mg for a single dose, as well as for 480 mg to 960 mg once a day with repeated administration for 5 days.

## Discussion

In this study, we confirmed the safety and tolerability of a single oral administration of ES16001 at concentrations of 240 to 1440 mg/kg and repeated oral administrations of 480 mg and 960 mg ES16001.

Since ES16001 has not been administered to humans, the starting dose was determined using the method for calculating the maximum recommended starting dose (MRSD) from the US FDA guideline “Estimating the Maximum Safe Starting Dose in Initial Clinical Trials for Therapeutics in Adult Healthy Volunteers”. The first step in estimating the MRSD proposed by the FDA guidelines is to convert the no observed adverse event level (NOAEL) calculated in the most appropriate or most sensitive animal species to the human equivalent dose (HED) taking into account the body surface area [14]. The doses used in the study were set based on the non-toxic doses identified in rats and beagles [13]. Repeated dose toxicity tests of the 50% ethanol extract of ES were performed over 13 weeks in rats and beagles, and the NOAELs were 1000 mg/kg and 500 mg/kg, respectively. After converting this to the HED, the maximum recommended starting doses (MRSD) calculated by applying the average adult weight (60 kg) and the safety factor (= 10) were 960 mg/day and 1620 mg/day from rats and beagles, respectively [13]. In addition, the pharmacologically active dose (PAD) in rats was 25 mg/kg, and the maximum was 100 mg/kg [13]. Considering the average weight of human adults (60 kg), the maximum PAD was 480 mg/day. Therefore, based on these results, 240 mg/day was determined as the starting dose.

To characterize ESE, quantitative analysis of geraniin, a major component of ESE, was performed via HPLC analysis with gradient elution. The content of geraniin in the 50% aqueous EtOH extracts was determined to be 152.68 μg/mg (Additional file 1: Figure S1). Although human pharmacokinetic (PK) analysis of ESE was not performed in this study, PK analysis based on the geraniin concentration in serum of rats administered orally with 1 g/kg of ESE calculated the C_max_ and T_max_ of geraniin in the serum at 0.013 mg/mL and 2 h, respectively. Also, the terminal elimination half-life (t_1/2_) of ESE was calculated as 7.43 h (Additional file 1: Figure S2). Human PK analysis will be performed together in a follow-up study to confirm efficacy in patients with herpes zoster.

In each dose group, subjects were randomized so that 6 subjects received oral ES16001 and 2 received oral placebo. The results of randomization were not disclosed to researchers or subjects through double blinding, and progression to the high-dose group was carried out step-by-step based on the safety and tolerability evaluation results of the previous dose group. During the clinical trial period, ALT elevation was observed as the most common ADR evaluated as ‘probably related’ or higher, and statistically significant AST and ALT elevations were observed as cohort 2 (480 mg single dose group) progressed. This was consistent with the characteristics of the investigational drug (AST, ALT elevation findings) observed in preclinical studies (beagle dogs, 500 mg/kg or 1000 mg/kg repeated administration for 4 weeks). Statistical analysis of liver function tests in all dose groups determined that the AST and ALT values according to time and dose showed a statistically significant upward trend. However, most of the test values are within the clinically insignificant range, and in most cases, it was judged to have little clinical significance on the basis of recovery to normal values immediately after the end of administration. The safety and tolerability of ES16001 was judged to be good because no abnormal findings or changes with clinical significance were observed in the results of other clinical laboratory tests, vital signs, physical examination, and electrocardiogram results.

## Conclusion

In conclusion, ES16001 was judged to have good safety and tolerability within the range of 240–1440 mg single dose and 5 repeated doses of 480 mg and 960 mg. Further research is needed to understand the possibility of drug-induced liver injury, which appeared infrequently. Our findings provide a rationale for further clinical investigations of ES16001. Based on the results of this study, follow-up studies are also needed to confirm the safety and efficacy in patients with herpes zoster.

## Supplementary Information

**Additional file 1:Table S1.** Virus particle number by ORF38 analysis for reactivation measurement in Vero cell using hollow fiber assay. **Figure S1.** HPLC results of the ESE. The LC chromatogram of the ESE (A) and geraniin (B). HPLC analysis was performed using a gradient elution on a Phenomenex Gemini-NX C18 column. The eluent consisted of 0.02% FA in H_2_O (A) and 0.02% FA in MeOH (B). The gradient profile (B) was as follows: 5% (0.01 min) → 5% (6 min) → 15% (10 min) → 20% (20 min) → 30% (30 min) → 70% (35 min) → 70% (45 min). UV absorption was measured at 220 nm, and the flow rate and column oven temperature were set at 1 mL/min and 30 °C, respectively. Quantitative analysis was replicated thrice. The chromatogram shown is representative of three independent experiments. **Figure S2.** HPLC chromatograms of serum (A) and concentration-time profile of geraniin (B) after oral administration of ESE at a dose of 1 g/kg in rat. Blood samples were centrifuged to obtain the serum at 7000 rpm under 4 °C for 20 min, and the supernatants of each sample were stored at − 20 °C until PK analysis. Each supernatant (1 mL) was mixed with equivalent MeOH (1 mL) and vortexed for 3 min. Then, each sample was centrifuged at 13,000 rpm under 4 °C for 20 min. Concentration of geraniin in serum was determined by a LC-20AD with a Skypack C18 column was used under gradient conditions. The eluent consisted of 0.1% phosphoric acid in H_2_O (A) and acetonitrile (B). The gradient profile (B) was as follows: 90% (0.01 min) → 90% (10 min) → 25% (12 min) → 90% (15 min). UV absorption was measured at 220 nm, and the flow rate and column oven temperature were set at 1 mL/min and 40 °C, respectively. The calibration curve (*y* = 53435793x+362690, *r*^2^ = 1.000) was linear in the range of 0.32–1000 ng/mL for geraniin.

## Data Availability

All data generated or analyzed during this study are included in the published article.
