# Supplementary material for: Safety, tolerability of ES16001, a novel varicella zoster virus reactivation inhibitor, in healthy adults
Source: Eur J Med Res. 2021 Aug 12;26:92. doi: 10.1186/s40001-021-00565-z (PMC8359576; doi:10.1186/s40001-021-00565-z)
Supplement: Supplementary file 1 — Additional file 1:Table S1. Virus particle number by ORF38 analysis for reactivation measurement in Vero cell using hollow fiber assay. Figure S1. HPLC results of the ESE. The LC chromatogram of the ESE (A) and geraniin (B). HPLC analysis was performed using a gradient elution on a Phenomenex Gemini-NX C18 column. The eluent consisted of 0.02% FA in H2O (A) and 0.02% FA in MeOH (B). The gradient profile (B) was as follows: 5% (0.01 min) → 5% (6 min) → 15% (10 min) → 20% (20 min) → 30% (30 min) → 70% (35 min) → 70% (45 min). UV absorption was measured at 220 nm, and the flow rate and column oven temperature were set at 1 mL/min and 30 °C, respectively. Quantitative analysis was replicated thrice. The chromatogram shown is representative of three independent experiments. Figure S2. HPLC chromatograms of serum (A) and concentration-time profile of geraniin (B) after oral administration of ESE at a dose of 1 g/kg in rat. Blood samples were centrifuged to obtain the serum at 7000 rpm under 4 °C for 20 min, and the supernatants of each sample were stored at − 20 °C until PK analysis. Each supernatant (1 mL) was mixed with equivalent MeOH (1 mL) and vortexed for 3 min. Then, each sample was centrifuged at 13,000 rpm under 4 °C for 20 min. Concentration of geraniin in serum was determined by a LC-20AD with a Skypack C18 column was used under gradient conditions. The eluent consisted of 0.1% phosphoric acid in H2O (A) and acetonitrile (B). The gradient profile (B) was as follows: 90% (0.01 min) → 90% (10 min) → 25% (12 min) → 90% (15 min). UV absorption was measured at 220 nm, and the flow rate and column oven temperature were set at 1 mL/min and 40 °C, respectively. The calibration curve (y = 53435793x+362690, r2 = 1.000) was linear in the range of 0.32–1000 ng/mL for geraniin. [file 40001_2021_565_MOESM1_ESM.docx]

**Safety, tolerability of ES16001, a novel varicella-zoster virus reactivation inhibitor, in healthy adults**

European Journal of Medical Research

Jeon Hwang-Bo^1, †^, Byungwook Kim^2, †^, Dae Won Park^1^, Yeong-Geun Lee^1^, Jeong Eun Kwon^1^, Jae-Yong Chung^3, *^ and Se Chan Kang^1, *^

^1^Graduate School of Biotechnology and Department of Oriental Medicine Biotechnology, Kyung Hee University, Yongin 17104, Republic of Korea

^2^Department of Clinical Pharmacology and Therapeutics, Seoul National University College of Medicine and Hospital, Seoul, Republic of Korea

^3^Department of Clinical Pharmacology and Therapeutics, Seoul National University College of Medicine and Bundang Hospital, Seongnam, Republic of Korea.

**^*^Correspondence to:** Se Chan Kang, [sckang@khu.ac.kr](mailto:sckang@khu.ac.kr); Jae-Yong Chung, [jychung@snubh.org](mailto:jychung@snubh.org)

| **Additional file 1: Table S1** Virus particle number by ORF38 analysis for reactivation measurement in Vero cell using hollow fiber assay. | | | |
| --- | --- | --- | --- |
| **Group** | **Virus particles number** | | **Reactivation rate (%)** |
|  | **1st** | **2nd** |  |
| Control (VZV infected Vero cells + PBS) | 8,006 ± 1076.5 | 9,280 ± 1,687.2 | 13.7 |
| VZV infected Vero cells + ESE 25 mg/kg | 51 ± 18.3 | 66 ± 20.6 | 22.0 |
| VZV infected Vero cells + ESE 50 mg/kg | 25 ± 3.5 | 24 ± 5.3 | -4.2 |
| VZV infected Vero cells + ESE 100 mg/kg | 23 ± 4.5 | 15 ± 2.9 | -60.3 |
| VZV infected Vero cells + Acyclovir 100 mg/kg | 210 ± 65.2 | 71 ± 13.3 | -196.1 |
| Data represented mean ± S.E. (n=5 and 3). ^**^*p*<0.01; significantly different from control. | | | |


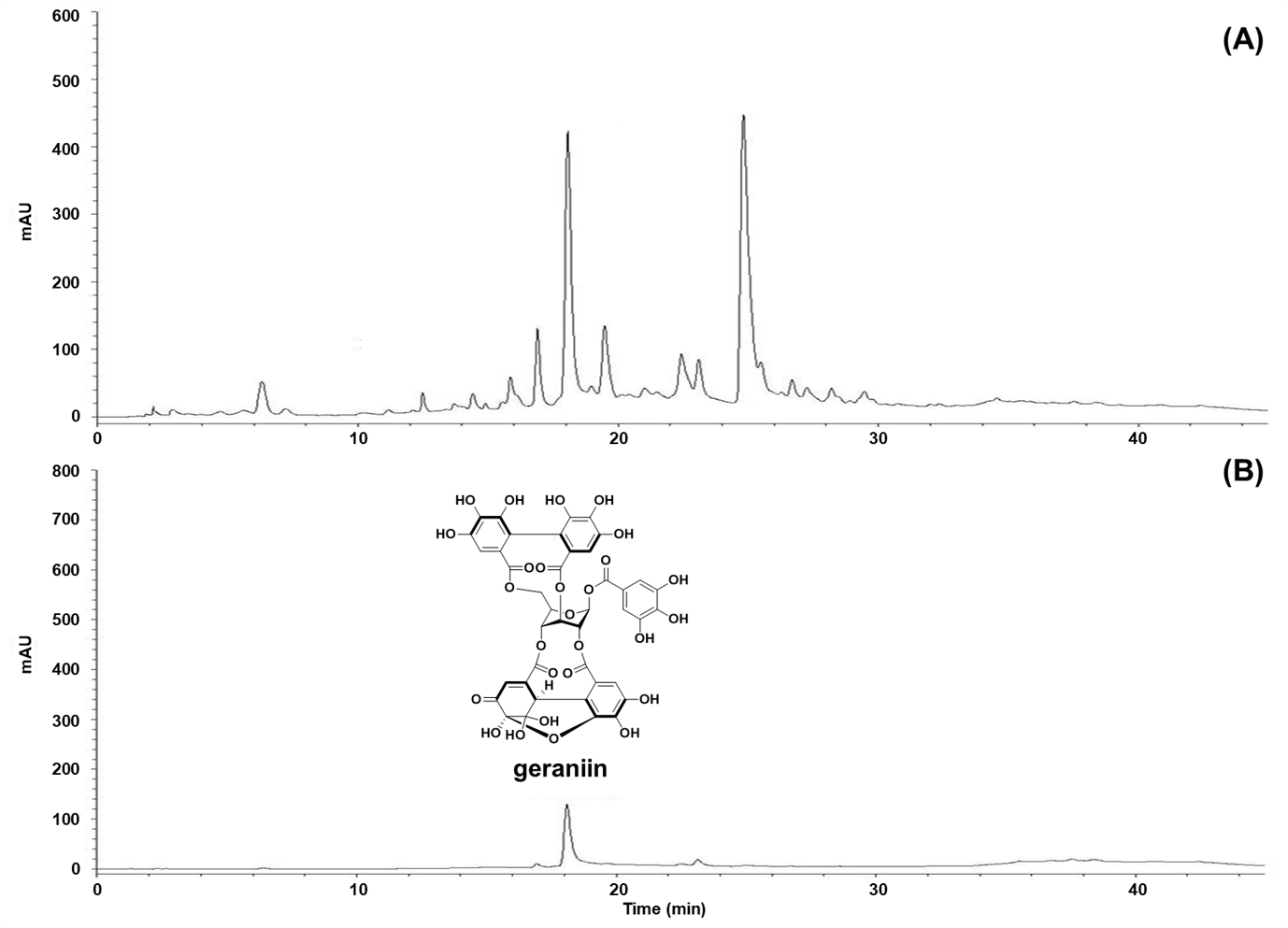


**Additional file 1: Figure S1** HPLC results of the ESE. The LC chromatogram of the ESE (A) and geraniin (B). HPLC analysis was performed using a gradient elution on a Phenomenex Gemini-NX C18 column. The eluent consisted of 0.02 % FA in H_2_O (A) and 0.02% FA in MeOH (B). The gradient profile (B) was as follows: 5% (0.01 min) → 5% (6 min) → 15% (10 min) → 20% (20 min) → 30% (30 min) → 70% (35 min) → 70% (45 min). UV absorption was measured at 220 nm, and the flow rate and column oven temperature were set at 1 mL/min and 30°C, respectively. Quantitative analysis was replicated thrice. The chromatogram shown is representative of three independent experiments.


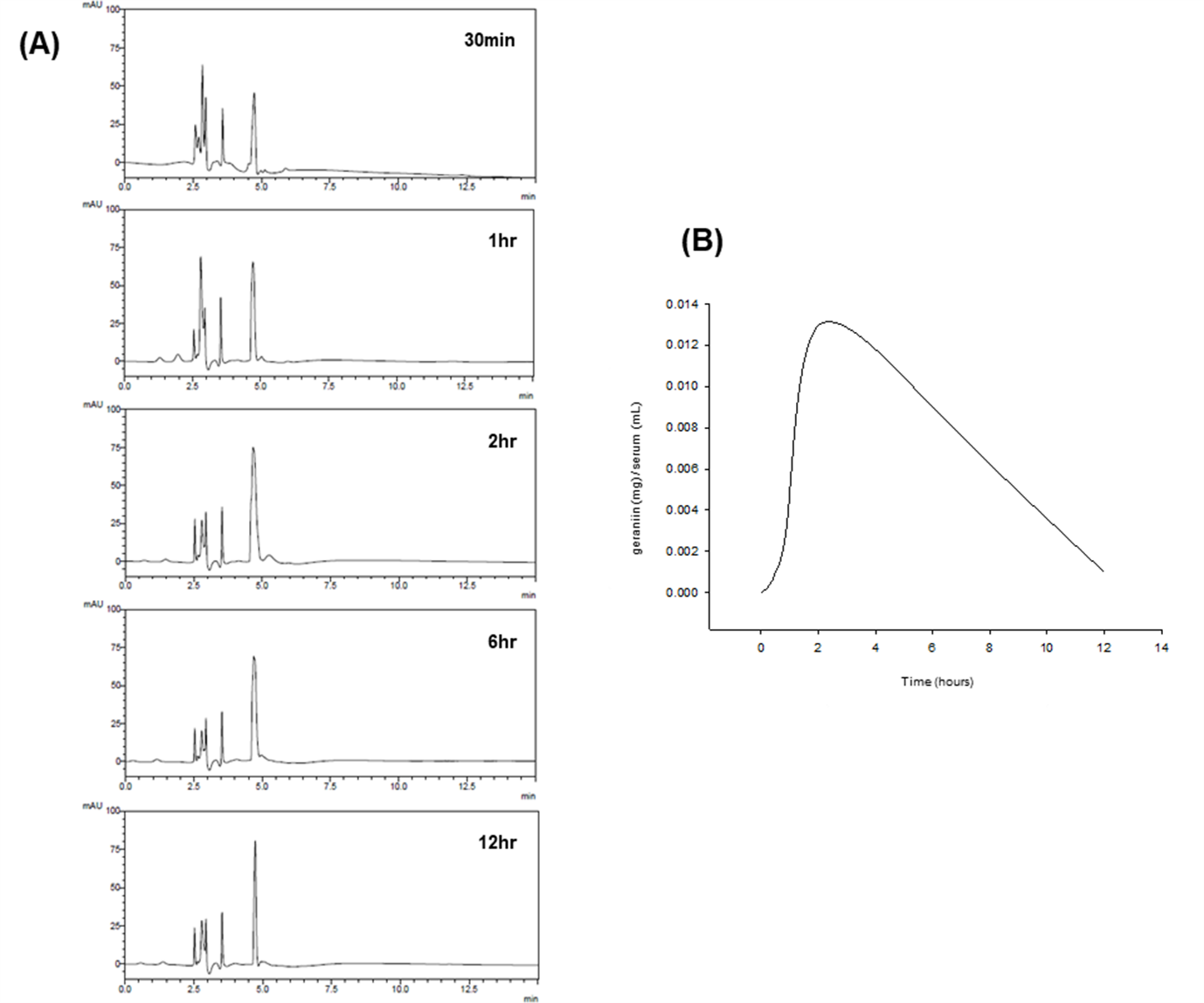


**Additional file 1: Figure S2** HPLC chromatograms of serum (A) and concentration-time profile of geraniin (B) after oral administration of ESE at a dose of 1 g/kg in rat. Blood samples were centrifuged to obtain the serum at 7,000 rpm under 4℃ for 20 min, and the supernatants of each sample were stored at -20°C until PK analysis. Each supernatant (1 mL) was mixed with equivalent MeOH (1 mL) and vortexed for 3 min. Then, each sample was centrifuged at 13,000 rpm under 4℃ for 20 min. Concentration of geraniin in serum was determined by a LC-20AD with a Skypack C18 column was used under gradient conditions. The eluent consisted of 0.1 % phosphoric acid in H_2_O (A) and acetonitrile (B). The gradient profile (B) was as follows: 90% (0.01 min) → 90% (10 min) → 25% (12 min) → 90% (15 min). UV absorption was measured at 220 nm, and the flow rate and column oven temperature were set at 1 mL/min and 40°C, respectively. The calibration curve (*y*=53435793*x*+362690, *r*^2^ = 1.000) was linear in the range of 0.32-1000 ng/mL for geraniin.
